# Qualitative development of the PROMIS Profile v1.0-Familial Chylomicronemia Syndrome (FCS) 28

**DOI:** 10.1007/s11136-022-03266-0

**Published:** 2022-10-31

**Authors:** Karen Kaiser, Rina S. Fox, Chelsea Perschon, Montserrat Vera-Llonch, Jordi Alonso, Laia Cubells, David Cella

**Affiliations:** 1 Northwestern University Feinberg School of Medicine, Chicago, IL USA; 2 University of Arizona College of Nursing, Tucson, AZ USA; 3 Ionis Pharmaceuticals, Carlsbad, CA USA; 4 Hospital del Mar Medical Research Institute, Barcelona, Spain; 5 Universitat Pompeu Fabra, Barcelona, Spain; 6 CIBERESP, CIBER in Epidemiology and Public Health, Madrid, Spain

**Keywords:** PROMIS, Rare disease, Familial chylomicronemia syndrome, Measure development, Patient report outcomes

## Abstract

**Purpose:**

Familial chylomicronemia syndrome (FCS) is a rare genetic disorder characterized by high triglyceride levels, significant disease burden, and negative impacts on health-related quality of life. This project aimed to create a PROMIS-based patient-reported outcome measure that represents valid and important concerns for patients with FCS.

**Methods:**

We reviewed the literature and data from a previous qualitative study of FCS to identify key FCS symptoms and impacts, which were mapped to PROMIS domains to create a pool of eligible items. Candidate items were reduced per expert feedback and patients with FCS completed cognitive interviews to confirm content validity and measure content.

**Results:**

Literature and qualitative data review identified ten key symptoms and 12 key impacts of FCS, including abdominal pain, fatigue, difficulty thinking, and worry about pancreatitis attacks. We identified 96 items primarily from PROMIS, supplemented with items from the Quality of Life in Neurological Disorders™ (Neuro-QoL™) and the Functional Assessment of Chronic Illness Therapy (FACIT) measurement systems. This pool was reduced to 32 candidate items, which were assessed via cognitive interviews with eight participants with FCS. Cognitive interview results and additional expert feedback led to the removal of four items and finalization of the PROMIS Profile v1.0—familial chylomicronemia syndrome (FCS) 28.

**Conclusions:**

The PROMIS Profile v1.0—familial chylomicronemia syndrome (FCS) 28 provides strong content validity for assessing quality of life among patients with FCS. The benefits of PROMIS, including norm-referenced mean values for each measure, will facilitate comparison of patients with FCS to other clinical populations.

## Introduction

Familial chylomicronemia syndrome (FCS) is a rare metabolic disorder characterized by high triglyceride levels and recurrent, severe bouts of acute pancreatitis [1, 2]. Despite significant, negative impacts of FCS on individual’s health-related quality of life (HRQOL), few detailed investigations of FCS symptoms and HRQOL are available [3]. The limited available data suggest a significant disease burden and several noteworthy symptoms and disease impacts, including pain, fatigue, brain fog, and stigma [3–11]. However, it remains unclear which symptoms are most impactful and important from the perspective of patients with FCS. Thus, additional work to identify the key symptoms and appropriate patient-reported outcome measures (PROMs) for FCS is needed.

Fox et al. [6] recently administered nine measures from The National Institutes of Health Patient-Reported Outcomes Measurement Information System® (PROMIS®) PROMIS and Quality of Life in Neurological Disorders™ (Neuro-QoL™) via an online survey to a sample of FCS patients and found that these measures captured worse global physical and mental health, anxiety, depression, physical function, fatigue, pain interference, cognitive function, and belly pain among individuals with FCS, relative to the general population. Moreover, FCS patients reported worse sleep disturbance, self-efficacy for managing social interactions and stigma relative to other chronic illness populations. Although generic PROMs provide opportunities to compare HRQOL across health conditions, a condition-specific measure of FCS symptoms and impacts could provide greater relevance to FCS, reduce respondent burden, and improve responsiveness [12–15]. Thus, this manuscript describes the development of an FCS-specific PROM from PROMIS. Given the unique features of PROMIS measures, including rigorous measure development [16], a norm-referenced mean value for each measure, and the use of item response theory (IRT), using a PROMIS measure for future HRQOL research among individuals with FCS has significant advantages.

## Methods

The approach for this project drew upon the methods used to develop and evaluate items pools for PROMIS [16], Food and Drug Administration guidance for the development of PROMs [17], and an approach for creating condition-specific measures from PROMIS developed by Schifferdecker et al. [14].

### Identification of key FCS symptoms and concerns

First, two Northwestern University (NU) researchers with extensive experience with PROMIS and PROM development independently reviewed concept elicitation interview transcripts and results from a previous study of FCS quality of life conducted with ten individuals with FCS in the United States (data on file, Ionis) [18]. The interviews consisted of open-ended questions about FCS symptoms, symptom frequency and severity, and impacts of FCS on daily life. The researchers met to discuss their impressions of the most important symptoms and impacts represented in the data and study report, and created a preliminary list of key symptoms and impacts for FCS.

The NU study team conducted a search in PubMed using the following terms: “(familial chylomicronemia syndrome) OR (lipoprotein lipase deficiency) OR (hyperlipoproteinemia type 1) AND (quality of life).” This search strategy was then adapted for Google Scholar. Relevant articles were extracted and reviewed by the team. The NU team also reviewed reference lists of extracted articles to ensure capture of key references. The study team reviewed the pertinent literature to confirm findings from their review of the qualitative transcripts and to consider whether there were additional symptoms and impacts central to the experience of living with FCS.

### Mapping FCS symptoms and concerns to PROMIS

Next, the team identified existing items from the PROMIS item banks, which currently contain over 1900 items, to represent symptoms and impacts included in the previous FCS study [18] and/or in the literature review. If no PROMIS items existed for a particular concept, other HealthMeasures measurement systems (https://www.healthmeasures.net/) were reviewed for possible items.

### Item reduction

The number of items in the pool was reduced via a series of meetings of the NU study team and joint meetings with FCS and patient-reported outcomes experts from Ionis. These meetings followed the PROMIS measure development methodology (i.e., the “item-review process”) and aimed to eliminate items that were redundant, confusing, poorly written, or did not adequately represent the symptoms and impacts identified in the patient data and literature [16, 19].

### Cognitive interviews

Although items drawn from PROMIS have undergone cognitive debriefing to evaluate language, comprehensibility, ambiguity, and relevance [16], we conducted cognitive interviews of the draft item set with individuals with FCS to confirm item clarity, meaning, and relevance for FCS. Participants for the cognitive interviews were recruited from a sample of individuals with FCS who participated in the 2018 study, “PROMIS®-Based Survey of Health-Related Quality of Life in Familial Chylomicronemia Syndrome.”[6]. All participants reported that they had a diagnosis of FCS, were 18 years of age or older, and lived in the United States. Participants who completed the PROMIS-Based Survey study (*N* = 25) were contacted via email and invited to participate in the telephone cognitive interview. Up to three recruitment emails were sent to each individual. Interested, eligible individuals completed informed consent, and the study coordinator scheduled their interview at a time that was convenient for them. Participants received a copy of the draft measure via email or postal mail prior to the interview.

Interviewers first collected sociodemographic and key disease information from the participant. Next, participants completed the draft measure and the interviewer led them through a series of questions about the measure, using a semi-structured cognitive interview guide based on the work of Willis [20] to ascertain comprehension of the measure items and the response processes. Specifically, the interviewer asked participants to: (1) describe how they arrived at their answer; (2) restate each item in their own words; (3) discuss the clarity of the item; (4) describe any questions they had about the item; and (5) indicate whether the question was relevant to their experience. Participants received a $100 USD electronic gift card for participating. Trained interviewers took detailed field notes, and interviews were audiotaped to ensure comprehensive capture of all relevant information. Cognitive interview recordings were transcribed and transcripts were de-identified. Transcripts were used to confirm field notes and to provide supporting quotations.

## Results

### Identification of key FCS symptoms and concerns

We identified nine key published articles about patient-reported symptoms and HRQOL in the context of FCS [3–11]. In the prior qualitative study by Davidson and colleagues, FCS interview participants reported 16 symptoms of FCS [18]. Of these, the most prevalent/important symptoms were abdominal pain, diarrhea, brain fog, and fatigue. Commonly reported symptoms per the key literature were abdominal pain, bloating, fatigue [5, 7]. Emotional, social and cognitive impacts of FCS in the literature included anxiety, cognitive difficulties, and work and social limitations [5, 7]. Based upon our review of the key literature and the Davidson article and data, we identified ten important FCS symptoms and 12 impacts, for a total of 22 key concepts (Table 1). The following symptoms, which were mentioned by patients in the prior qualitative study, were excluded from our list of the most import symptoms: blurred vision, poor appetite, difficulty concentrating, weight loss, indigestion, muscle weakness. The 12 impacts shown in Table 1 expand upon the findings by Davidson by, for example, detailing specific impacts related to mental and emotional well-being and adding the concept of sleep disruption [6].

**Table 1:** Important symptoms and impacts for FCS patient QOL

Concepts identified from existing data and literature	Initial item pool (96 items)		After item reduction (32 items)	
	# of Items	Source(s)	# of Items	Source(s)
Symptoms				
Abdominal pain	5	PROMIS	5	PROMIS
Diarrhea	6	PROMIS	6	PROMIS
Difficulty thinking	6	PROMIS, Neuro-QoL	6	PROMIS, Neuro-QoL
Physical fatigue	12	PROMIS	12	PROMIS
Bloating	11	PROMIS	11	PROMIS
Nausea	1	PROMIS	1	PROMIS
Vomiting	1	PROMIS	1	PROMIS
Pain (not abdominal)	5	PROMIS	5	PROMIS
Xanthomas	2	PROMIS, Neuro-QoL	2	PROMIS, Neuro-QoL
Difficulty remembering words, names	9	PROMIS	9	PROMIS
Impacts				
Daily functioning				
Dietary restrictions	–	–	–	–
Physical activity	3	PROMIS	3	PROMIS
Sleep disturbance	4	PROMIS	1	PROMIS
Social functioning				
Social activities and planning	4	PROMIS	2	PROMIS
Ability to work/volunteer				
Productivity	2	PROMIS	2	PROMIS
Negative career impact	5	FACIT	0	–
Financial				
Financial strain	1	FACIT	1	FACIT
Mental/emotional well-being				
Worry about pancreatitis attack	5	PROMIS	1	Neuro-QoL
Judged because of diagnosis	4	PROMIS, Neuro-QoL	1	Neuro-QoL
Future health worries	2	Neuro-QoL	1	Neuro-QoL
Burden to others	3	PROMIS, Neuro-QoL	1	Neuro-QoL
Depressed because of diagnosis	5	PROMIS, ASCQ-Me	1	PROMIS

### Mapping FCS symptoms and concerns to PROMIS

Next, existing items (*N* = 96) were identified to represent the 22 key concepts. Existing items were identified for all symptoms and for every impact except “impact of dietary restrictions,” as this concept does not fall within the scope of HRQOL as measured by HealthMeasures. Thus, this concept was excluded. Identified items representing the remaining 21 key concepts (ten symptoms and eleven impacts) originated from PROMIS, Neuro-QoL, and the Adult Sickle Cell Quality of Life Measurement System (ASCQ-Me®), which is also a HealthMeasures measurement system. An item addressing financial strain was draw from the Functional Assessment of Chronic Illness Therapy (FACIT) measurement system [21].

### Item reduction

A series of investigator meetings were held to reduce the item pool. Item retention decisions were based on identifying items that best fit the FCS symptoms and impacts and provided a variety of response options for key concepts (e.g., frequency, intensity, and interference). When there were comparable choices, preference was given to items from the PROMIS 29 + 2 Profile v2.1. The PROMIS 29 + 2 has been included in other FCS clinical trials, using items from the measure would therefore reduce response burden and ensure inclusion of items that have been tested and used extensively [22, 23]. The team reviewed the following information for each concept: definition of the concept, priority of the concept for inclusion in the measure, article/data supporting the concept, draft items and the source, time frame, and response option for each draft item. The team discussed which item(s) best fit each concept, as well as whether alternate items should be sent to cognitive interviewing. For example, five items in the pool covered the concept of abdominal pain. Of these, three items were retained. The item, “How often did you have discomfort in your belly?” was dropped in favor of items that used the word “pain” as pain was more consistent with the literature and data. The item “How much did belly pain bother you?” was dropped in favor of items on belly pain frequency, worst pain, and interference in day-to-day activities. Using this general approach, the 96 items were reduced to 32 items. These 32 items became the draft measure presented to participants in the cognitive interviews. Additionally, the concept “negative career impact” was dropped as the concept was outside the HRQOL of HealthMeasures, leaving 20 key concepts.

### Cognitive interviews

Twelve individuals responded to our email request. Of these, two declined to participate, one was ineligible, and one did not attend their scheduled interview. Cognitive interviews were completed with eight unrelated individuals with FCS. The cognitive interview sample characteristics (*N* = 8) are shown in Table 2. The sample was primarily female (*n* = 6, 75.0%). All participants indicated that they followed an FCS diet at least some of the time. Six participants (75.0%) were experiencing symptoms of FCS at the time of the interview. The mean number of self-reported acute pancreatitis attacks over the last 5 years was ten (range 0–50).

**Table 2:** Cognitive interview sample characteristics (*N* = 8)

Patient characteristics	
Mean age in years (range)	46.25 (25–64)

	% (*n*)
Gender	
Female	75.0% (6)
Male	25.0% (2)
Race/ethnicity	
White	100.0% (8)
Education	
High school graduate/GED	12.5% (1)
Some college/technical degree/AA	37.5% (3)
College degree (BA/BS)	37.5% (3)
Advanced degree (MA, MS, MBA, PhD, MD, JD)	12.5% (1)
Marital status	
Currently married	75.0% (6)
Single (never married)	25.0% (2)
Employment status	
Employed full time	50.0% (4)
Homemaker	12.5% (1)
Retired	12.5% (1)
Unemployed	12.5% (1)
On disability	12.5% (1)
Health insurance	
Private insurance	87.5% (7)
Medicaid	12.5% (1)
Activity level	
Normal activity without symptoms	25.0% (2)
Some symptoms but do not require rest	50.0% (4)
Require bed rest for < 50% of waking day	25.0% (2)
Require bed rest for > 50% of waking day	0.0% (0)
Follows an FCS diet…	
All the time	12.5% (1)
Most of the time	62.5% (5)
Sometimes	25.0% (2)
Rarely	0.0% (0)
Never	0.0% (0)
Ever experienced acute pancreatitis	
Yes	100.0% (8)
Experienced recurrent pancreatitis^a^	
Yes	75.0% (6)
Diabetes diagnosis	
Yes	75.0% (6)
No	25.0% (2)

	Mean (range)
Age when FCS symptoms began (years)	17.6 (0–42)
Age at time of FCS diagnosis (years)	37.1 (0–60)
Number of acute pancreatitis attacks in past 5 years	10.0 (0–50)
Typical daily fat intake (grams) (*n* = 6)^b^	15.0 (10–30)
Age when diagnosed with diabetes (years) (*n* = 6)^c^	29.8 (13–45)

^a^Recurrent pancreatitis was defined as having more than one acute pancreatitis attack in the last 5 years

^b^Participants who did not know their daily fat intake (*n* = 2) were coded as missing

^c^Participants who did not have diabetes (*n* = 2) were coded as missing

### Instructions, response options, and length of questionnaire

Every cognitive interview participant (*N* = 8, 100%) said that the measure instructions were clear and the overall length of the questionnaire was about right. Likewise, when asked, “These questions ask you to respond using several different response options. Did these response options make sense to you?” all 8 participants said yes. When asked if it was easy to respond using the response options, all 8 participants said yes. (Items and response options presented to participants in the cognitive interviews are available from the first author.)

### Face validity

Face validity was assessed with the following interview question: “Please take a moment to look over the questions again. Do these questions, in your opinion, capture your experiences with FCS?” All 8 participants answered yes to this question.

### Content validity

Content validity was assessed in two ways. For each item on the measure, participants were asked, “What kinds of things did you think about when you answered the question?” Responses were analyzed to determine whether participants were interpreting the items in ways that were consistent with intended item meanings. None of the items were found to be misinterpreted by participants.

Content validity was also assessed by asking participants if other important questions about FCS were missing from the questionnaire. Most participants (*n* = 7, 87.5%) said there were questions missing from the questionnaire. Three participants cited managing one’s diet as important missing content. However, because dietary restriction is outside the scope of the HRQOL definition, no items were added to reflect this concept. Two participants mentioned educating doctors about FCS, and one participant mentioned knowledge of triglycerides and connections to other people with FCS. These concepts are also outside the scope of our HRQOL definition. One participant said that there should be a specific item about xanthoma pain. The team agreed that xanthoma-specific pain would be a challenging attribution for a patient to make, and that the general pain item would suffice. Thus, no changes were made to the draft measure based on this feedback.

### Respondent understanding of the items

For 30 of the 32 draft items, every participant said the meaning of the item was clear. For the item, “I have trouble thinking clearly,” participant 002 said they thought the item could be made clearer by specifying trouble thinking due to FCS. For the item, “I have trouble doing all of my usual work (include work at home),” participant 004 thought that the meaning of work from home was confusing because so many people are working from home due to the COVID-19 pandemic. The participant suggested using the phrase “household chores” or “domestic things” instead of “work from home.” No changes were made due to these comments.

### Item preferences: difficulty thinking

The draft measure included 5 items related to difficulty thinking (Table 3). Participants were asked which of the items best fit their FCS experience. The most frequently chosen item was, “My thinking has been slow,” which was chosen by four participants (50.0%). The next most commonly selected item was, “I have been able to concentrate,” which was selected by three participants (37.5%). The item, “I have been able to remember to do things, like take medicine or buy something I need,” was selected least often (*n* = 1, 12.5%).

**Table 3:** Participant preference for difficulty thinking items (*N* = 8)

Item	Participant ID								Total
	01	02	03	04	05^a^	06	07	08	
My thinking has been slow		X	X	X		X			4
I have been able to concentrate	X						X	X	3
I have trouble thinking clearly	X					X			2
I have had trouble recalling the name of an object while talking to someone		X	X						2
I have been able to remember to do things, like take medicine or buy something I need	X								1

X = Item chosen as best fitting their experience

^a^Participant 05 did not endorse any of the items about difficulty thinking because they did not experience cognitive problems

### Item preferences: worry

The draft item set included two items regarding worry about one’s health (Table 4). Participants preferred the item, “I worry that my condition will get worse” (seven participants vs. five participants). Four participants did not prefer one item over the other and noted that useful, unique information could be obtained from each item.

**Table 4:** Participant preference for worry items

Item	Participant ID								Total
	01^a^	02	03	04	05	06	07	08	
I worry that my condition will get worse		X	X	X	X	X	X	X	7
I worried about my physical health	X			X	X	X		X	5

^a^Participant 01 preferred the physical health item, but noted that it would be okay to have both items

### Measure finalization

Following cognitive interviews, the investigator team met three times to finalize the measure. During these meetings, the team reviewed the 20 key concepts and cognitive interview results. Twenty-eight items were retained for the FCS measure and four items were removed. For each item removed, alternate items were preferred by the study team and/or cognitive interview participants (Table 5). When possible, items from the PROMIS 29 + 2 measure were utilized so as to reduce participant burden in situations where the PROMIS 29 + 2 is being used in research or clinical settings.

**Table 5:** Items removed from final PROMIS FCS measure

Draft item	Symptom/impact assessed	Reason for removal
I had trouble thinking clearly	Difficulty Thinking	Team and cognitive interview participants preferred “My thinking has been slow” and “I have been able to concentrate.” The latter item is on the PROMIS 29 + 2
I have had trouble recalling the name of an object while talking to someone	Difficulty remembering (words, names)	Team preferred “I have been able to remember to do things, like take medicine or buy something I need.” This item is on the PROMIS 29 + 2
I worried about my physical health	Worry about pancreatitis attack	Team and cognitive interview participants preferred “I worry that my condition will get worse”
How much difficulty do you have doing your physical activities, because of your health?	Physical activity	Team preferred the following items to assess physical function and exercise: “To what extent are you able to carry out your everyday physical activities such as walking, climbing stairs, carrying groceries, or moving a chair?” and “Does your health now limit you in exercising regularly?”

### Final measure

The PROMIS Profile v1.0—familial chylomicronemia syndrome (FCS) 28 (PROMIS FCS 28) is shown in Table 6. The measure contains the most important symptoms for FCS (16 items, first column), including abdominal pain (three items), pain (two items), fatigue (two items), and brain fog or cognitive difficulties (three items). Given the importance of pain to FCS patients, the PROMIS FCS 28 assesses the frequency of, intensity of, and interference due to abdominal pain. Additionally, key impacts of FCS are including in the measure (12 items, second column), such as worry (one item), impacts on social activities (two items), physical activity (two items), and productivity (two items).

**Table 6:** Final items in the PROMIS profile v1.0—familial chylomicronemia syndrome (FCS) 28^a^

Symptoms	Impacts
How often did you have belly pain?	Because of my illness, I worried about other people’s attitudes towards me
At its worst, how would you rate your belly pain?	Because of my illness, I worried that I was a burden to others
How much did belly pain interfere with your day-to-day activities?	I worry that my condition will get worse
I feel fatigued	I felt depressed
I have trouble starting things because I am tired	My sleep quality was…
How many days did you have loose or watery stools?	I have trouble doing all of the activities with friends that I want to do
How much did loose or watery stools interfere with your day-to-day activities?	I have trouble doing all of the family activities that I want to do
My thinking has been slow	I have trouble doing all of my usual work (include work at home)
I have been able to concentrate	I have trouble doing everything for work that I want to do (include work from home)
I have been able to remember to do things, like take medicine or buy something I need	To what extent are you able to carry out your everyday physical activities such as walking, climbing stairs, carrying groceries, or moving a chair?
I was unhappy about how my illness affected my appearance	Does your health now limit you in exercising regularly?
How often did you feel bloated?	My illness has been a financial hardship to my family and me
How often did you have nausea—that is, a feeling like you could vomit?	
How often did you throw up or vomit?	
How much did pain interfere with your day-to-day activities?	
How would you rate your pain on average?	

^a^The PROMIS FCS 28 with response options for each item is available from the first author

## Discussion

Using the recently developed method for creating condition-specific PROMIS measures [14], we identified key symptoms and impacts of FCS and created a pool of items from PROMIS and other domains to represent those concerns. A team with extensive experience in measure development and FCS reduced the item pool to a set of items that were confirmed as relevant and clear by a sample of FCS patients. As such, we have completed the first step towards the development of a specific FCS PRO with the appropriate content. Future work can assess the reliability and validity of this new measure in FCS patients. Prior work using this method of adapting PROMIS has produced measures with good psychometric properties [15] and we anticipate the same will be true of the PROMIS FCS 28.

The PROMIS and Neuro-QoL items that make up the PROMIS FCS 28 have been calibrated to a common metric. This facilitates comparability across various studies and populations; this may be especially important for a rare disease such as FCS. Moreover, by selecting items reflecting the most relevant symptoms and impacts for FCS, the PROMIS FCS 28 provides a tailored measure that reduces the response burden on participants while gathering rich HRQOL data on key symptoms such as abdominal pain, cognitive difficulties, fatigue, and impacts on social activities. For example, because abdominal pain can be both chronic and episodic for individuals with FCS [7], the measure captures pain frequency, intensity, and interference. This nuanced patient-reported information is especially important in rare diseases such as FCS, where clinicians and researchers may be especially reliant on reports of the disease and its impact from the patient.

Participants in this study were demographically similar to patients with FCS who participated in past research. For example, in the APPROACH study [24], the largest known study to date among patients with FCS (*N* = 66), the mean age was 46 years (range 20 to 75), and the majority of the sample was female (55%) and White (80%). For the prior qualitative study of FCS quality of life from which key FCS symptoms and concerns were initially identified [18], the mean age was 53 years (range 28 to 69), and the majority of the sample was female (70%) and White (100%).

FCS can be difficult to distinguish from multifactorial chylomicronemia and patients may be misdiagnosed. Several aspects of our study increase our confidence that our cognitive interview participants were correctly diagnosed with FCS. First, over two years passed between recruitment into the Fox study [6] and recruitment into our study (July/August 2018 to November/December 2020). This time lapse provides an opportunity for a misdiagnosis to be identified and corrected. Second, the high rate of pancreatitis in our sample—every participant had experienced pancreatitis and six of the eight participants (75%) had multiple attacks of acute pancreatitis in the last five years—is more consistent with FCS than with multifactorial chylomicronemia [25].

### Limitations

While the broad applicability of a PROMIS-based measure increases its reach and relevance, developing a condition-specific measure from PROMIS does present certain limitations. For example, whereas PROMIS domains include a wide-range of content, we were limited to the existing item wording and response options. However, cognitive debriefing showed that the chosen items worked well for FCS—their meaning was clear, relevant for FCS, and the response options worked well. Future work can evaluate the extent to which assessment of dietary intake and career impact add additional value. Additionally, the investigator team did not include anyone living with FCS. As such, the methods utilized may not have targeted the needs and priorities of FCS patients as optimally as possible. Another limitation is that the sample was relatively homogenous and the response rate to recruitment attempts was approximately 50%. Although participants were demographically similar to those who participated in past studies of patients with FCS, it is possible that results may have been impacted by selection bias, thus limiting generalizability. Finally, the psychometric properties of the PROMIS FCS 28 were not evaluated. Given the rare nature of FCS, the scientifically strong development of this measure represents notable progress toward the appropriate assessment of patients living with this disease and will enable improved understanding of the patient experience. Additionally, as outlined above, this approach for adapting PROMIS measures has yielded measures with strong psychometric properties in other populations, suggesting the same will be true of patients with FCS. However, it will nonetheless be important to evaluate the reliability and validity of this condition-specific tool in patients with FCS.

## Conclusion

While recent qualitative and quantitative work has grown our understanding of the disease burden of FCS, additional work, particularly quantitative assessments using valid patient-reported outcome measures, are needed to build our understanding of FCS, and to aid clinicians in caring for FCS patients [26]. Utilizing existing PROMIS items facilitates uptake of condition-specific measures into routine clinical practice and electronic health record systems [15]. Moreover, because there are currently no FDA-approved medications for FCS, the PROMIS FCS 28 fills a critical need for clinical trials of FCS therapies.

## Data Availability

N/A.

## Code availability

N/A.
